# Pseudanthia in angiosperms: a review

**DOI:** 10.1093/aob/mcad103

**Published:** 2023-07-21

**Authors:** Jakub Baczyński, Regine Claßen-Bockhoff

**Affiliations:** Institute of Evolutionary Biology, Faculty of Biology, Biological and Chemical Research Centre, University of Warsaw, Warsaw, Poland; Department of Plant Biology, Miller Plant Sciences, University of Georgia, Athens, Georgia, USA; Institute of Organismic and Molecular Evolution, Johannes Gutenberg-University, Mainz, Germany

**Keywords:** Aggregation, development, division of labour, evolution, floral unit, flowering plants, genetic regulation, inflorescence, meristem conditions, pollination biology, ray flower, showy bract

## Abstract

**Background:**

Pseudanthia or ‘false flowers’ are multiflowered units that resemble solitary flowers in form and function. Over the last century the term ‘pseudanthium’ has been applied to a wide array of morphologically divergent blossoms, ranging from those with easily noticeable florets to derived, reduced units in which individual flowers become almost indistinguishable. Although initially admired mostly by botanists, the diversity and widespread distribution of pseudanthia across angiosperms has already made them a fascinating topic for evolutionary and developmental comparative studies.

**Scope:**

This review synthesizes historical and current concepts on the biology of pseudanthia. Our first aim is to establish a clear, operational definition of pseudanthium and disentangle common terminological misconceptions surrounding that term. Our second aim is to summarize knowledge of the morphological and developmental diversity of pseudanthia and embed it within a modern phylogenetic framework. Lastly, we want to provide a comprehensive overview on the evolution and ecological importance of pseudanthia and outline perspectives for future studies.

**Conclusions:**

The understanding of pseudanthia has changed multiple times and reflects three different interpretations of their ‘flower-like’ qualities: developmental (similarity in structure), figural (similarity in form and function) and phylogenetic (homology between angiosperm flowers and monoecious reproductive shoots in gymnosperms). Here, we propose to narrow the term *pseudanthium* to multiflowered blossoms resembling zoophilous flowers in form, i.e. in being structurally subdivided in a showy periphery and a reproductive centre. According to this definition, pseudanthia *sensu stricto* evolved independently in at least 41 angiosperm families. The recurrent acquisition of pseudanthia *sensu stricto* in all major lineages of flowering plants indicates repeated interactions between developmental constraints (smallness of flowers, meristematic conditions) and selective pressures, such as demands of pollinators and/or environmental conditions.

## INTRODUCTION

### A historical perspective on pseudanthia

The fact that *multiflowered blossoms* can mimic the appearance of a single flower was already well recognized by the dawn of modern botany. In *Methodus Plantarum Nova*, John Ray (1682) for the first time referred to the capitulum of Asteraceae as a *flos compositus* (compound flower), according to a pharmaceutical convention making no distinction between true flowers and flower-heads (i.e. *Matricariae flos* = head of chamomile, see Drobnik, 2022). The same term for capitula was later adopted by Linné (1792), while Ehrhart (1787), on the other hand, considered it misleading; for him, proceeding from a synthetical rather than analytical perspective, calling a multiflowered structure a ‘compound flower’ seemed like ‘calling a group of people a compound human’. Instead, he re-adopted the term *anthodium* for blossoms of which *form* resembles a solitary flower (originally used by Linnaeus in 1770 to describe the involucre of Asteraceae). The use of *anthodium* in that sense was later corroborated in the works of Stokes (1812), Link (1837), Hanstein (1882) and Johow (1884).

The proper term *pseudanzio* was first introduced by the Italian botanist Federico Delpino (1889), one of the co-founders of pollination biology. Delpino was particularly interested in flowers in which the arrangement of floral organs broke the rules of phyllotaxy (i.e. with obdiplostemony or stamen fascicles). He postulated that these were in fact contracted inflorescences and, thus, ‘false flowers’ (*pseudanzia*), compared to ‘true flowers’ or *euanzia*. This difference in *structure* served as the basis for his new classification of angiosperms (Delpino, 1890, 1892): *Pseudante* included spurges (*Euphorbia* L., Euphorbiaceae), the cyathia of which constitute a single reproductive unit consisting of highly reduced flowers, but also Rosaceae or Malvaceae with stamen fascicles, while *Euante* united plants with ‘conventional’ flowers.

Almost 20 years later, Richard von Wettstein (1907) applied the term pseudanthium to multi-axial reproductive units found in gnetalean gymnosperms and proposed that angiosperm flowers evolved from such structures, based on the assumption of the close relationship between those two groups. This hypothesis, frequently referred to as ‘pseudanthial theory of flower origin’, remained highly influential throughout the 20^th^ century (Melville, 1960; Meeuse, 1972; Crane, 1985; Doyle, 1994; Krassilov, 1997; Sun *et al.*, 2002) and served as alternative to the euanthial theory (Arber and Parkin, 1907; Doyle and Donoghue, 1986), under which flowers were interpreted as simple uniaxial systems similar to those of cycads and extinct Bennettitales. The ideas of von Wettstein added *phylogenetic* context to a belief that at least some angiosperm flowers might be derived from ‘inflorescence-like’ structures. Although the pseudanthial theory became untenable with the demise of the anthophyte hypothesis (assuming a sister relationship between angiosperms and Gnetales, see Donoghue and Doyle, 2000), it is sometimes evoked in the light of relatively recent studies suggesting that strobili in cordaites, conifers and Gnetales may be derived from ancestral multiaxial structures (Mundry and Stützel, 2004; Rudall and Bateman, 2010).

Apart from von Wettstein, the current understanding of pseudanthia was strongly influenced by Wilhelm Troll (1928, 1964), a famous plant morphologist who was heavily rooted in German *Naturphilosophie* (Nickel, 1996; Weberling, 1999; Claßen-Bockhoff, 2001; Rieppel, 2011). His belief in idealistic morphology understood as an immanent urge to a certain form (*Gestalt*, understood as the manifestation of a given type) found its support in the study of asteracean capitula. Pseudanthia *sensu* Troll were correspondingly flower-like *forms* realized on the material level of an inflorescence. As multiflowered blossoms with clearly distinguishable individual florets, they include the same examples as *anthodia sensu* Ehrhart (Claßen-Bockhoff, 1991a).

Troll also postulated that the transition from an inflorescence to a pseudanthium may have occurred in three steps: (1) aggregation and miniaturization of flowers, (2) formation of *pseudocorollas* through differentiation of peripheral flowers and (3) co-option of extrafloral organs. This sequence served as inspiration for various theories on the morphological evolution of pseudanthia (Froebe and Ulrich, 1979; Claßen-Bockhoff, 1990; Harris, 1999); however, it is important to note that Troll’s view was purely typological and disconnected from a phylogenetic and evolutionary perspective (Claßen-Bockhoff, 2001).

Ronald Good (1956) came across pseudanthia *sensu* Troll during his systematic studies. For him, pseudanthia ‘function as and simulate a single true flower’ (p. 276); they evolved due to a general *aggregation tendency* in nature leading to *evolutionary repetitions*. However, the increasing association of flowers is based only on aggregation until it reaches a certain threshold defined by spatial conditions. Beyond this ‘last extreme state, there is, over and beyond any mere aggregation of the flowers, such a measure of arrangement, organization and division of labour [ … ] that the inflorescence as a whole attains a design and function greater than any that may result simply from the proximity or contiguity of units all like one another’ (Good 1956: 276). By considering developmental constraints triggering pseudanthia formation he paved the way for an *evo-devo* understanding of pseudanthia.

Elmar Emil Leppik presented yet another interesting perspective on pseudanthia. While primarily focusing on *form* (similarly to Ehrhart, Troll and Good), his approach encompassed ecological and evolutionary aspects. Based on studies on the pollination biology of Asteraceae, Leppik (1959, 1970) formulated the hypothesis of *pseudanthic recapitulation*, which implies that densely aggregated flower-heads not only increase general attraction, but also repeat well-established evolutionary patterns in response to pollinator-mediated selection. Next to an ‘*unexplained genetic mechanism*’ (Leppik 1959) reducing flower size to a minimum, he saw co-evolution between floral patterns and pollinator sensory perceptions as the two main evolutionary forces triggering pseudanthia formation. Like Good (1956), Leppik had the view that the entire pseudanthium can serve as an equivalent of a single pollination unit.

Regine Claßen-Bockhoff (1990, 1991a, 1992) provided a comprehensive summary of pseudanthia (*sensu* Troll) in angiosperms. She distinguished four types: floral vs. hyperfloral pseudanthia based on the purely floral constitution of the blossom vs. integration of extrafloral elements, and undifferentiated vs. differentiated blossoms dependent on the absence vs. presence of a pseudocorolla surrounding the reproductive centre. She reconstructed the morphological changes from inflorescences to pseudanthia within selected lineages and demonstrated that pseudanthia only differed gradually from inflorescences making a clear definition almost impossible. She concluded that aggregation, miniaturization and colouring might be the result of inhibition processes ranging from internode inhibition and developmental simplification to inhibition of chlorophyll synthesis. Ontogenetic abbreviations (neoteny) thus appear to play a major role also expressed by the persistence of juvenile stages and, most importantly, pattern repetition on the multi-flowered level. Beyond heterochrony, spatial constraints (ray flower formation) and shifts in function (from protective to showy organs) may promote flower similarity. Summarizing, the parallel evolution of flower-like patterns was assumed to be driven by a combination of lineage-specific phylogenetic traits, combined with developmental options and selective pressures.

At the beginning of the 21^st^ century, Paula Rudall introduced the term *fuzzy pseudanthia* to describe peculiar reproductive units in which florets become so highly reduced that the boundaries between flower and inflorescence become indistinct or blurred. Initially, she identified this pattern in cyathia (Prenner and Rudall, 2007) and in reproductive units of Hydatellaceae (Rudall *et al.*, 2009), which, based on ontogenetic data, were interpreted as ‘hybrid structures’. Later, fuzzy pseudanthia were also applied to reproductive structures of mycoheterotrophic Triuridaceae, including bizarre inside-out flowers (with centrally located stamens that are surrounded by carpels) of *Lacandonia schismatica* E.Martínez & Ramos (Triuridaceae, Rudall *et al.*, 2016) and staminal fascicles of *Ricinus communis* L. (Euphorbiaceae, Prenner *et al.*, 2008), re-interpreted as multiflowered floral units by Claßen-Bockhoff and Frankenhäuser (2020). The idea of an imprecise inflorescence–flower boundary is also clearly visible in her earlier studies on Pandanales (Rudall and Bateman, 2006). Having *structure* as a background, fuzzy pseudanthia approach closely to pseudanthia *sensu* Delpino. The situation in which an uncommon flower reveals itself as a multifloral unit upon closer inspection is also applicable to *synanthia*, which, according to Mattfeld (1938), gave rise to monoecious units of some sedges (Cyperaceae).

### Definition of pseudanthia

To analyse the diversity of pseudanthia one must adopt a precise definition of what is and – more importantly – what is not the real subject of the study. Following historical meanings of the term, the most logical choice would be to focus on structure and development, but such data are rarely available for most pseudanthia. The ecological definition of pseudanthia (*inflorescence-blossoms sensu*Claßen-Bockhoff, 1991a) is also problematic, as it requires making the distinction between *pollination unit* and *attraction unit* (Faegri and Pijl, 1979). Without a sharp ecological definition of pseudanthia, we are left with *anthodia* (pseudanthia *sensu* Troll) or blossoms the *form* of which resembles that of a single flower. But what does exactly floral *form* mean? Being aware that a sharp separation of pseudanthia from modified inflorescences is almost impossible, we take the heteromorphic heads of composites for the most unambiguous examples of the expression of a floral *Gestalt* in multifloral units. We thus focus on structurally subdivided pseudanthia (*sensu*Claßen-Bockhoff, 1990) and define them as *clearly multiflowered blossoms divided into a central part that serves reproductive functions and peripheral, usually radiating advertising/protective structures consisting of (1) distinctly enlarged, often sterile peripheral flowers and/or (2) showy, coloured extrafloral organs (e.g. bracts, prophylls, stem leaves*).

**Fig. 1.**
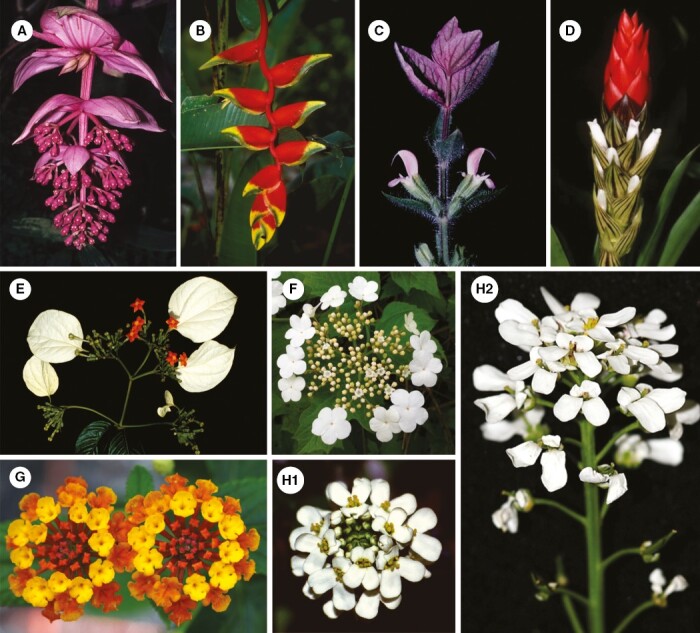
Modified inflorescences. (A, B) Inflorescences with showy bracts lacking aggregation: (A) *Medinilla magnifica* Lindl. (Melastomataceae); (B) *Heliconia rostrata* Ruiz & Pavon (Heliconiaceae). (C, D) Tuft blossoms: (C) *Salvia viridis* L. (Lamiaceae); (D) *Guzmania monostachia* (L.) Rusby ex Mez, (Bromeliaceae). (E, F) Inflorescences with ray flowers lacking aggregation: € *Mussaenda frondosa* L. (Rubiaceae); (F) *Viburnum opulus* L. (Adoxaceae). (G, H) Temporary flower-like inflorescences: (G) *Lantana camara* L. (Verbenaceae); (H) *Iberis amara* L. (Brassicaceae): young (H1) and older, elongated raceme (H2). Photos: B–E from Claßen-Bockhoff (2023).

**Fig. 2.**
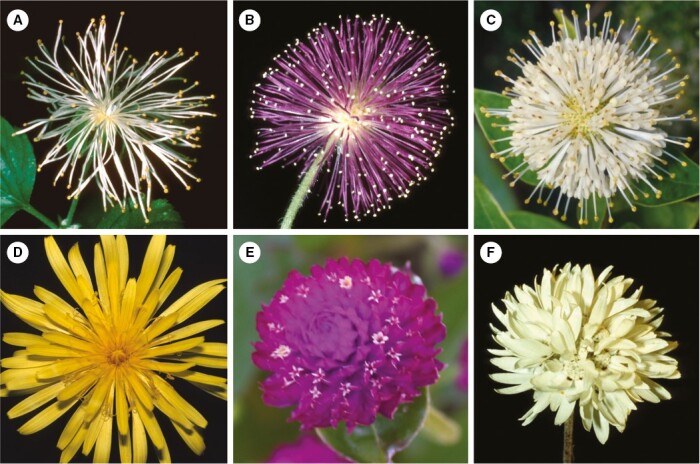
Pseudanthia *sensu lato*, lacking structural subdivision. (A–C) Pincushion-blossoms: (A) *Neviusia alabamensis* (Rosaceae) – flower with many stamens, included for comparative reasons; (B) *Mimosa pudica* (Fabaceae) – about 100 flowers, each with four stamens and one style; (C) *Cephalanthus occidentalis* (Rubiaceae) – thyrse-like floral unit (head with cymes) with many flowers, each secondarily presenting pollen on its style. (D) Homomorphic heads: *Taraxacum officinale* (Asteraceae). All flowers zygomorphic. (E, F) Pseudanthia *s.l.* with showy extrafloral elements: (E) *Gomphrena globosa* (Amaranthaceae) – minute flowers, each with two violet prohylls; (F) *Cephalipterum drummondii* (Asterraceae) – head-like raceme with many capitula, each with a showy white involucrum. Photos: A–C from Claßen-Bockhoff (2023).

This definition does not encompass modified inflorescences which share only a few flower-like characters (Fig. 1). Such inflorescences may have showy bracts below (Fig. 1A, B) or above the flowers (tuft blossoms, Fig. 1C, D), or enlarged single florets (Fig. 1E, F), but lack aggregation and smallness of flowers. Inflorescences with a transitory flower-like appearance due to flowering sequence (Fig. 1G) or delayed internode elongation (Fig. 1H) do not match our pseudanthium definition, too. Contrary to Claßen-Bockhoff (1990), we do not consider pseudanthia that are not structurally subdivided into centre and periphery (Fig. 2). They are characterized by flowers which lost their individuality by becoming part of a pincushion blossom (Fig. 2B, C), polymerous disc-blossom (Fig. 2D) or globular blossom (Fig. 2E, F). These types of blossoms may resemble a single flower (Fig. 2A), but lack the subdivision into reproductive field and surrounding showy structures. It is important to note that while homomorphic heads of some Asteraceae (Fig. 2D) do not fall under our definition of pseudanthia *sensu stricto*, others, such as those of *Tragopogon pratensis* L. (with distinctly enlarged peripheral ray flowers) or *Cichorium intybus* L. (with sexual centre formed by stamens and carpels of ray flowers and showy margin consisting of their ligules), may do so. Finally, reproductive units for which similarity to flowers is only reflected in development are also excluded, i.e. those that fulfil the definition of pseudanthia *sensu* Delpino or fuzzy pseudanthia.

## DIVERSITY OF PSEUDANTHIA

Pseudanthia *sensu stricto*, i.e. the structurally subdivided forms of pseudanthia (referred to simply as pseudanthia in the following paragraphs), show an immense diversity that results from the evolutionary history of various angiosperm lineages and specific morphological and ecological constraints.

### Phylogenetic distribution of pseudanthia

**Fig. 3.**
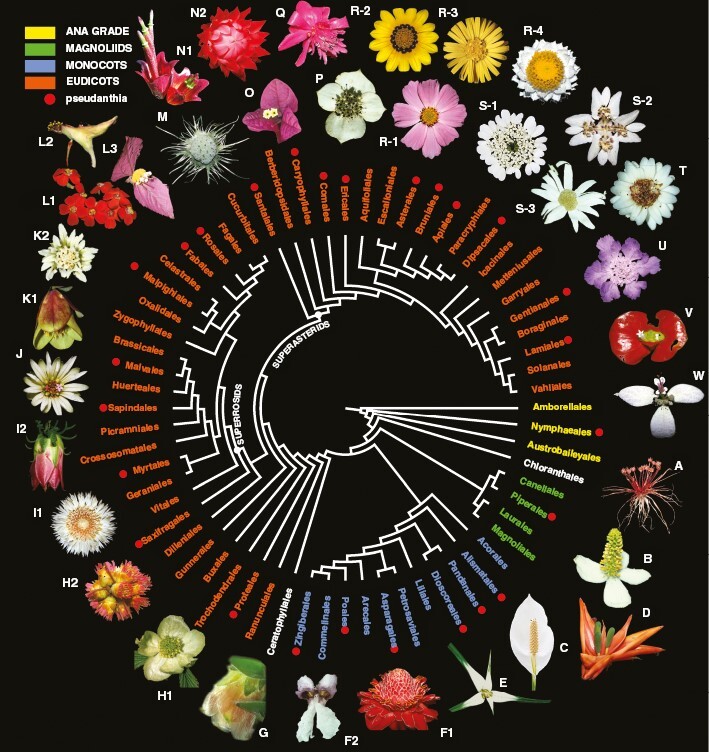
Phylogenetic diversity of pseudanthia *sensu stricto*. ANA grade. (A) Nymphaeales: *Trithuria submersa* (Hydatellaceae). Magnoliids. (B) Piperales: *Houttuynia cordata* (Saururaceae). Monocots. (C) Alismatales: *Spathiphyllum cochlearispathum* (Araceae). (D) Pandanales: *Freycinetia* sp. (Pandanaceae). (E) Poales: *Dichromena ciliata* (Cyperaceae). (F) Zingiberales: (1) *Etlingera elatior* (Zingiberaceae), (2) *Thalia geniculata* (Marantaceae). Basal eudicots. (G) Proteales: *Orothamnus zeyheri* (Proteaceae). Superrosids. (H) Saxifragales: (1) *Parrotiopsis jaquemontiana* (Hamamelidaceae), (2) *Rhodoleia championi* (Hamamelidaceae). (I) Myrtales: (1) *Darwinia lejostyla* (Myrtaceae), (2) *Actinodium cunninghamii* (Myrtaceae). (J) Sapindales: *Euchaetis longibracteata* (Rutaceae). (K) Malvales: (1) *Pimelea physodes* (Thymeleaceae), (2) *Lasiopetalum discolor* (Malvaceae). (L) Malpighiales: (1) *Euphorbia fulgens* (Euphorbiaceae), (2*) Euphorbia* (sect. *Pedilanthus*) *macrocarpa* (Euphorbiaceae), (3) *Dalechampia spathulata* (Euphorbiaceae). (M) Rosales: *Dorstenia yambuyaensis* (Moraceae). Superasterids. (N) Santalales: (1) *Tolypanthus lagenifer* (Loranthaceae), (2) *Thonningia sanguinea* (Balanophoraceae). (O) Caryophyllales: *Bougainvillea glabra* (Nyctaginaceae). (P) Cornales: *Cornus canadensis* (Cornaceae). (Q) Ericales: *Cavendishia adenophora* (Ericaceae). (R) Asterales: (1) *Cosmos bipinnatus* (Asteraceae), (2) *Gazania rigens* (Asteraceae), (3) *Eroeda capensis* (Asteraceae), (4) *Ammobium alatum* (Asteraceae). (S) Apiales: (1) *Artedia squamata* (Apiacaeae), (2) *Xanthosia rotundifolia* (Apiaceae), (3) *Actinotus leucocephalus* (Apiaceae). (T) Bruniales: *Staavia dodii* (Bruniaceae). (U) Dipsacales: *Scabiosa* sp. (Caprifoliaceae). (V) Gentianales: *Palicourea* sp. (Rubiaceae). (W) Lamiales: *Congea velutina* (Lamiaceae). Cladogram based on Li *et al.* (2019). Photos: A, Kevin Thiele, Wikimedia Commons. C, J.J. Harrison, Wikimedia Commons. N1, Vinyaraj, Wikimedia Commons. N2, KAKPO Sunday Berlioz, Wikimedia Commons. Q, Marcelo Aguilar, Wikimedia Commons.

Pseudanthia can be found in at least 41 plant families (Claßen, 1984; Claßen-Bockhoff, 1990; Table 1). However, they have never been referred to the current phylogenetic framework, hindering us from drawing firm conclusions about macroevolutionary trends. Herein, we provide a simple juxtaposition of pseudanthial diversity onto the most recent angiosperm phylogenetic tree (Chase *et al.*, 2016), which reveals some interesting patterns (Fig. 3).

**Table 1.** List of families with pseudanthia *sensu stricto*

Systematics	Morphology	Ov.	References
**ANA Grade**			
**Hydatellaceae** *Trithuria* Hook.f.	Minute head (FU) with bracts, monoclinous flowers reduced to a single stamen or carpel; carpellate florets usually surround the staminate ones	1	Hamann 1998 **Rudall *et al.* 2009**
**Magnoliids**			
**Saururaceae** *Houttuynia cordata* Thunb. *Anemopsis californica* (Nutt.) Hook. & Arn.	Dense spike with white bracts, flowers monoclinous, staminate at the tip of the inflorescence	6–16	Tucker 1981 **Tucker 1985** Cheng-Yih and Kubitzki 1993
**Monocotyledons**			
**Bromeliaceae** *Canistrum* É.Morren *Edmundoa* Leme *Wittrockia superba* Lindm. *Wittrockia cyathiformis* (Vell.) Leme	Simple or compound racemes with white or coloured bracts	num	Smith and Till 1998 Santos-Silva *et al.* 2017 Nogueira *et al.* 2021
**Cyperaceae** *Rhynchosphora* (L.) Vahl., sev. spec.	Cluster of spikelets with few white bracts	1	**Leppik 1955** Goetghebeur 1998 **da Costa *et al.* 2021**
*Ficinia radiata* (L.f.) Kunth	Cluster of spikelets with many yellow bracts		**Claßen-Bockhoff 1990** Goetghebeur 1998
**Eriocaulaceae, e.g.** *Eriocaulon xeranthemum* Mart. *Syngonanthus* Ruhland *Comanthera* L.B.Sm. *Mesanthemum* Körn. *Paepalanthus* Mart., sev. spec.	Head (FU) with many white or yellow bracts, flowers monoclinous, staminate florets in the median part of the head	2–3	**Hensold, 1988** Stützel, 1998 **Stützel and Trovó 2013**
**Poaceae** *Sokinochloa australis* S. Dransf.	Cluster of spikelets with brownish bracts	1	Kellogg 2015 Dransfield 2016
**Pontederiaceae** *Heteranthera* (syn. *Hydrothrix) Gardneri* (Hook.f.) M.Pell.	Pair of zygmorphic flowers forming an actinomorphic, hexamerous blossom	num	**Rutishauser 1983** Cook 1998
**Marantaceae** *Thalia* L.	Pair of asymmetric flowers forming a bilabiate blossom with showy staminodes	1	**Claßen-Bockhoff 1991*b*** Andersson 1998 **Dworaczek and Claßen-Bockhoff 2016**
**Zingiberaceae** *Etlingera* (syn. *Nicolaia*) *elatior* (Jack) R.M.Sm.	Head with many red bracts	num	**Claßen 1987** Larsen *et al.* 1998
**Amaryllidaceae** *Haemanthus* L. sev. spec. *Scadoxus* Raf. sev. spec.	Umbel-like cymoid with coloured spathe bracts	3–6	Müller-Doblies and Müller-Doblies 1975 Meerow and Snijman 1998 Martínez-Gómez *et al.* 2022
**Orchidaceae** *Rhizanthella gardneri* R.S.Rogers	Head with white bracts that serve protective function (all species are mycoheterotrophic and bloom underground)	num	**Balogh 1982** **Dixon *et al.* 1990**
**Colchicaceae** *Colchicum* L. sev. spec. (syn. *Androcymbium* Willd.)	Few-flowered head with two white or reddish bracts	num	**Vogel 1963** Nordenstam 1998 Manning *et al.* 2007
**Dioscoreaceae** *Tacca* J.R.Forst. & G.Forst. sev. spec.	cymoid with ≥2 brown large bracts and many dark filamentous prophylls	num	Huber 1998 Zhang *et al.* 2011 **Chua *et al.* 2020** **Martínez-Gómez *et al.* 2022**
**Pandanaceae** *Freycinetia* Gaudich, sev. spec.	Compound spadix with coloured, fleshy bracts, flowers monoclinous, blossoms dioecious	num	Cox 1990 Stone *et al.* 1998
**Triuridaceae (?)** *Lacandonia schismatica*. E.Martínez & Ramos	Minute head with radiating bracts, highly reduced female flowers surround male ones; alternatively interpreted as a flower with inside-out morphology	1	**Rudall 2003** **Ambrose *et al.* 2006**
*Sciaphila* Blume *Triuris* Miers *Triuridopsis* H.Maas & Maas	Dioecious heads with radiating bracts; alternatively interpreted as monoclinous flowers	1	**Rudall 2003**; **Ambrose *et al.* 2006**
**Araceae several genera, e.g. *Philodendron* Schott.**	spadix (FU) with spatha, flowers monoklinous or perfect	1 to num	**Vogel 1963** **Gottsberger and Amaral 1984** Mayo *et al.* 1998 **Barabé and Lacroix 2008b**
**Aponogetonaceae** *Aponogeton distachyos*	Spike; white flower-subtending bracts floating on the water surface	num	Bruggen 1998
**Eudicotyledons**			
**Proteaceae,** e.g. *Orothamnus* Pappe ex Hook. *Serruria* Burm. ex Salisb.	Head with coloured bracts, cup- or bell-shaped	1 to num	Weston 2007
**Hamamelidaceae** *Parrotiopsis jacquemontiana* (Decne.) Rehder	Head (FU) with two bistipulate bracts forming a hexamerous blossom	2	**Claßen-Bockhoff 1990** Endress 1993 **Claßen-Bockhoff and Bull-Hereñu 2013**
*Rhodoleia* Champ. ex Hook.	Head with red, zygomorphic flowers, the peripheral petals of them enlarged	num	**Claßen-Bockhoff 1990** Endress 1993 **Gu *et al.* 2010**
**Rhamnaceae** *Phylica plumosa* L. *Stenanthemum pimeleoides* (Hook.f.) Benth. *Siegfriedia darwinioides* C.A.Gardner *Spyridium* Fenzl sev. spec.	Head-like cymes or thyrses with white showy bracts	2–8	**Claßen-Bockhoff 1990** Medan and Schirarend 2004
**Moraceae** *Dorstenia* L. sev. spec.	Head-like FU* (‘cymoid’) with a collar of filamentous bracts, monoclinous flowers united in a monoecious pseudanthium	1	**Bernbeck 1932** Rohwer and Berg 1993 Leite 2016
*Antiaropsis* K.Schum.	Head-like unit with red bracts		
**Peraceae** *Pera* Mutis	FU (‘composed cyme’) with bracts; plant dioecious with distinct staminate and carpellate pseudanthia	3	Webster 2014 **Gagliardi *et al.* 2018**
**Phyllanthaceae** *Uapaca* Baill	Head subtended by large creamy-yellowish bracts, plant dioecious	6	McPherson 2011 Webster 2014
**Euphorbiaceae**, e.g. *Euphorbia milii* L.	Cyathium (FU) with two red prophylls	3–6	**Claßen-Bockhoff 1990** Webster 2014
*Euphorbia heterophylla* L.	Cyathium (FU) with (partially) red leaves		
*Euphorbia fulgens* Karw. ex Klotzsch	Cyathium (FU) with five red, stipular excrescences, actinomorphic blossom		**Michaelis 1924** **Prenner and Rudall 2007** Webster 2014
*Euphorbia macrocarpa* Boiss & Buhse.	Cyathium (FU) with five red, stipular excrescences, zygomorphic		
*Neoguillauminia cleopatra* (Baill.) Croizat	FU (‘flower/inflorescence hybrid’) with white stipular excrescences		**Prenner and Rudall 2007** Webster 2014
*Dalechampia spathulata* (Scheidw.) Baill.	FU (? ‘complex sciadoid’) with two coloured prophylls; staminate cyme surrounded by carpellate cymes		**Froebe *et al.* 1983** Webster 2014
**Fabaceae** *Neptunia* Lour.	Head with peripheral perfect flowers and a centre consisting of by staminate and carpellate flowers	6	**Tucker 1988**
*Calliandra surinamesis* Benth.	Pincushion-blossom with few nectar flowers in the centre having elongated androecial tubes		**Claßen 1984**
**Myrtaceae** *Darwinia* Rudge sev. gen.	Bell-shaped head (FU?) with reddish bracts	2–10	**Claßen-Bockhoff 1992** Wilson 2011
*Actinodium cunninghamii* Schauer	Proliferating head (inflorescence) with showy short shoots (white bracts and prophylls)	1	Wilson 2011 **Claßen-Bockhoff *et al.* 2013**
**Malvaceae** *Lasiopetalum* Sm. sev. spec.	Cymoid with whitish hairy bracts	6–8	**Claßen 1988** Bayer and Kubitzki 2003
**Rutaceae** *Diplolaena* R.Br. sev. spec.	FU (‘reduced panicle’) with reddish bracts	sev.	**Claßen-Bockhoff *et al.* 1991** Kubitzki *et al.* 2011
*Euchaetis longibracteata* Schltr.	Flowering shoot system with single flowers in basipetal order* and white bracts		Kubitzki *et al.* 2011
**Thymelaeaceae** *Pimelea physodes* Hook	Bell-shaped head with reddish bracts	1	**Claßen-Bockhoff 1992** Beaumont *et al.* 2001 Herber 2003
*Pimelea sulphurea* Meisn. *Gnidia somalensis* (Franch.) Gilg	Head with bright yellow bracts		
**Balanophoraceae** *Langsdorffia* Mart. *Thonningia* Vahl,	Head with coloured bracts; flowers monoclinous, plant dioecious (rarely monoecious)	nn	Hansen 1980
**Loranthaceae** *Tolypanthus* (Blume) Rchb.	Few-flowered head with fused, coloured bracts	nn	Dixit 1961 **Kuijt 1981** Kuijt and Hansen 2015
**Nyctaginaceae** *Bougainvillea* Comm. ex Juss.	Triad (‘partial cymose inflorescence’) with three coloured bracts	1	Bittrich and Kühn 1993 **Xu *et al.* 2009**
*Allionia* L.	Triad (‘partial cymose inflorescence’) of zygomorphic flowers		Bittrich and Kühn 1993 Richman and Venable 2018
**Nyssaceae** *Davidia involucrata* Baill.	Head (FU) with two white, flag-like bracts; andromonoecious with a single perfect flower or completely staminate	6–10	Kubitzki 2004 **Claßen-Bockhoff and Arndt 2018**
**Cornaceae** *Cornus* L. sev. spec.	FU* (‘paniculate cyme’, ‘minidichasium’, ‘thyrso-panicle’) with four white or pinkish bracts; flowers rarely monoclinous	1–2	Kubitzki 2004 **Feng *et al.* 2011**
**Asteraceae**, e.g. *Ammobium alatum* R. Br.	Head (FU) with white involucral bracts	1	Anderberg *et al.* 2007 **Claßen-Bockhoff 1996a**
*Polycalymma stuartii* F.Muell. & Sond. ex F.Muell. & Sond.	Pleiochasial cymoid with heads (FUs) surrounded by white bracts		
*Myriocephalus guerinae* F. Muell.	Head (FU) of headlets (FUs) with secondary receptacle and white bracts		
*Cosmos bipinnatus* Cav.	Head (FU) with zygomorphic, carpellate ray flowers; plant gynomonoecious		**Troll 1928** Anderberg *et al.* 2007
*Centaurea jacea* L.	Head (FU) with radial, perfect ray flowers		
*Tragopogon pratensis* L.	Homomorphic head (FU) with peripheral ray flowers being distinctly larger than the central ones		
*Dyssodia decipiens* (Bartl.) M.C.Johnst. ex M.C.Johnst. & L.Turner	Botryoid composed of heads (FU) with ray flowers only on the outer heads; plants gynomonoecious		**Strother 1977** **Claßen-Bockhoff 1992**, **1996*a*** Anderberg *et al.* 2007
*Oedera capensis* Druce			
**Bruniaceae** *Staavia dodii* Bolus *Staavia glutinosa* (P.J.Bergius) Dahl	Flowering shoot system with single flowers in basipetal order and white bracts	1	**Claßen-Bockhoff 2000** Claßen-Bockhoff 2016 Anderberg *et al.* 2007
*Brunia paleacea* P.J.Bergius	Head (FU?) with wwhite bracts	1–2	
**Caprifoliaceae** *Scabiosa* L. *Knautia* L.	Head (FU*) with zygomorphic ray flowers	1	Carlson *et al.* 2011 Hofmann and Bittrich 2016
**Ericaceae** *Cavendishia Adenophora* Mansf. *Cavendishia nitens* Sleumer	Axillary, head-like racemose inflorescence with pinkish bracts	num	Luteyn 1983 Stevens *et al.* 2004
**Apiaceae,** e.g.: *Xanthosia* Rudge	Umbel (FU) of umbellets (FUs) with ≥4 white involucellar bracts, plant andromonoecious	2	**Froebe, 1979** Plunkett *et al.* 2018
*Actinotus* Labill. *Alepidea* F. Delaroche *Astrantia* L.	Capitate FU with several whitish involucral bracts, plants andromonoecious		**Froebe 1964**, **1979** Plunkett *et al.* 2018
*Echinophora trichophylla* Sm. *Artedia squamata* L. *Coriandrum sativum* L.	Umbel (FU) of umbellets (FUs), zygomorphic ray flowers, plants andromonoecious		**Froebe 1980** Plunkett *et al.* 2018 **Baczyński *et al.* 2022**
**Rubiaceae** *Psychotria* (incl. *Cephaelis)* L., *Palicourea* Aubl. sev. spec.	Thyrse (FU?) with ≥4 coloured bracts, flowers perfect, rarely monoclinous	2	**Claßen-Bockhoff 1996*b***
*Stipularia africana* P. Beauv.	Cymoid enclosed by stipulate bracts	num	
**Lamiaceae** *Congea* Roxb. *Symphorema* Roxb. *Sphenodesme* Jack	Cyme with flower-subtending bracts, tetramerous or trimerous (after bract fusion)	4	**Claßen 1985** Harley *et al.* 2004

Systematics: after APG IV 2017. Morphology: cymoid is defined as a simple, cymosely branched unit. FU, floral units; question marks indicate probable FUs that require further investigation. *Unpubl. data. sev. spec., several species. Ov, ovules per flower; nn, not known (due to extreme flower reduction); num, numerous. Quotation marks indicate original morphological terms used in references. References: selection of references referring to the morphology and/or patterning of the pseudanthia (bold type) and to systematic aspects, respectively.

Pseudanthia occur in all major clades of flowering plants (Table 1). They were described at the base of the angiosperm phylogeny (Fig. 3A) in Hydatellaceae (Rudall *et al.* 2009), mycoheterotrophic representatives of which produce inconspicuous, predominantly self-pollinated blossoms (Taylor *et al.*, 2010). Among magnoliids, flower-like inflorescences are characteristic of Saururaceae (Hutchinson, 1926), notably the genera *Houttuynia* Thunb. (Tucker, 1981; Fig. 3B) and *Anemopsis* Hook. & Arn. (Tucker, 1985). In monocots, inflorescence-blossoms originated multiple times (Fig. 3C–F) but are relatively infrequent. Apart from Araceae (Fig. 3C), none of the species-rich families described within this clade can be considered entirely or predominantly pseudanthial while in most of them such architecture is restricted to only a few representatives. The greatest architectural variety of pseudanthia is unequivocally associated with eudicots. In some lineages, only single and isolated species are pseudanthial, such as in Rutaceae (*Eucheaetis longibracteata* Schltr.; Fig. 3J) or Hamamelidaceae (*Parrotiopsis jacquemontiana* Decne., *Rhodoleia championi* Hook.; Fig. 3H1, H2), whereas in others pseudanthia are ubiquitous, as in Euphorbiaceae (with *Euphorbia* including ~2000 species, Fig. 3L), Rubiaceae (Fig. 3V) and Apiaceae (Fig. 3S). Occasionally, pseudanthia may even constitute a major synapomorphy, an example being the largest angiosperm family Asteraceae with ~20 000 species (Fig. 3R).

### Morphological diversity of showy elements

**Fig. 4.**
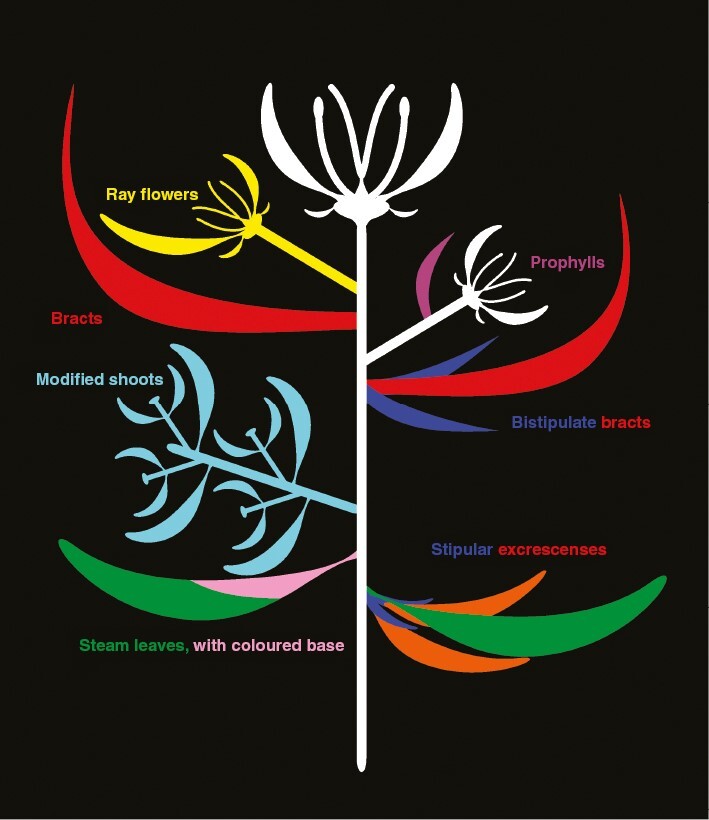
Diversity of showy organs in pseudanthia *sensu stricto*. The scheme shows a three-flowered inflorescence with the main petaloid elements. Colours: blue – stipules, green – stem leaf (pink indicating petaloid area), orange – stipular excrescences, red – flower-subtending bract, turquoise – modified lateral shoot, violet – prophyll, yellow – ray flower.

**Fig. 5.**
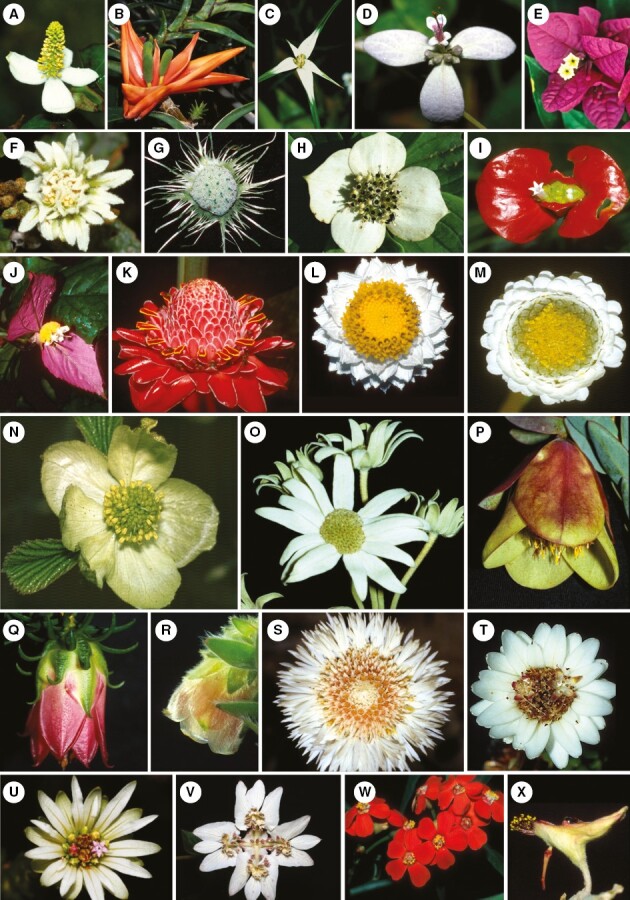
Pseudanthia *sensu stricto* with showy extrafloral elements. (A) Spike with showy bracts: *Houttuynia cordata* (Saururaceae). (B) Three spadices with showy bracts: *Freycinetia* sp. (Pandanaceae). (C) Cluster of spikelets with showy bracts: *Dichromena ciliata* (Cyperaceae). (D) Cyme with showy bracts: *Congea velutina* (Lamiaceae). (E) Three-flowered unit: *Bougainvillea glabra* (Nyctaginaceae). (F) Cymoid with showy bracts: *Lasiopetalum discolor* (Malvaceae). (G) Cymoid-like head with showy bracts: *Dorstenia yambuyaensis* (Moraceae). (H, I) Thyrse-like head with showy bracts: (H) *Cornus canadensis* (Cornaceae); (I) *Palicourea* sp. (Rubiaceae). (J) Thyrse-like head with showy prophylls: *Dalechampia spathulata* (Euphorbiaceae). (K, L) Heads with showy bracts: (K) *Etlingera elatior* (Zingiberaceae); (L) *Ammobium alatum* (Asteraceae). (M) Heads of headlets with showy bracts: *Myriocephalus helichrysoides* (Asteraceae). (N) Head with showy bistipulate bracts: *Parrotiopsis jacquemontiana* (Hamamelidaceae). (O) Head-like floral units with showy bracts: *Actinotus helianthi* (Apiaceae). (P–R) Bell-shaped heads with showy bracts: (P) *Pimelea physodes* (Thymeleaeaceae); (Q) *Darwinia leiostyla* (Myrtaceae); (R) *Orothamnus zeyheri* (Proteaceae). (S) Head with modified short-shoots: *Actinodium cunninghamii*. (T, U) Clusters of single flowers with showy bracts: (T) *Staavia doddii* (Bruniaceae); (U) *Euchaetis longibracteata* (Rutaceae). (V) Umbel of umbellets with showy involucellar bracts: *Xanthosia rotundifolia* (Apiaceae). (W, X) Cyathia with showy stipular excrescences (Euphorbiaceae): (W) *Euphorbia fulgens*; (X) *Euphorbia macrocarpa*. A–P, R, T–W from -Claßen-Bockhoff (2023).

Perianth-like structures, which serve as the basis for the definition of the pseudanthium *sensu stricto*, can develop from almost every type of organ (Fig. 4; Table 1 and citations therein). Co-option of bracts is probably the most widespread architectural solution. Bracts are defined as phyllomes that appear below or within the flowering zone and differ from the green (frondose) leaves in being smaller and inconspicuous or larger and showy. They are usually simple in shape and inserted with a broad base, emphasizing their frequent function as protective elements. Some are sterile [as in *Etlingera elatior* (Jack) R.M.Sm (Fig. 5K)], others subtend a flower (Fig. 4: red, Fig. 5D), a partial inflorescence (Fig. 5F) or hold a prophyll position (Fig. 4: violet, Fig. 5J). Prophylls are filiform in the blossoms of some *Tacca* J.R.Forst. & G.Forst. (Taccaceae) and *Dorstenia* Plum. ex L. (Moraceae) species (Fig. 5G). Stipules contribute to the hexamerous pattern in *Parrotiopsis jacquemontiana* (Decne.) Rehder (Fig. 4: blue, Fig. 5N; Claßen-Bockhoff, 1992), fused prophylls to the trimery of *Congea velutina* Wight (Lamiaceae; Fig. 5D; Claßen, 1985), and asymmetrical involucellar bracts to an umbel-centred arrangement of white elements in *Xanthosia rotundifolia* DC. (Apiaceae; Fig. 5V; Froebe, 1979). Stipular excrescences form actinomorphic and zygomorphic blossoms in the genus *Euphorbia* (Fig. 4: orange, Fig. 5W, X; Michaelis, 1924) and short shoots with several white bracts and prophylls surround the flower-head in the pseudanthium of *Actinodium cunninghamii* Schauer ex Lindl. (Myrtaceae; Fig. 4: turquoise, Fig. 5S; Claßen-Bockhoff *et al.*, 2013). Pseudanthia with showy bracts are either polymerous (Fig. 5T) or have a fixed organ number (Fig. 5H). Both patterns usually reflect the phyllotaxis of the vegetative shoot system. Showy bracts can be white (e.g. Fig. 5A, M, V), sometimes due to a dense pubescence (Fig. 5D, F, O), or coloured, with red and reddish as dominant tones (e.g. Fig. 5B, I, K). Few species show a gradual colouring of their bracts with white or red spots at the base of otherwise green leaves (i.e. *Euphorbia heterophylla* L., Fig. 4: pink).

**Fig. 6.**
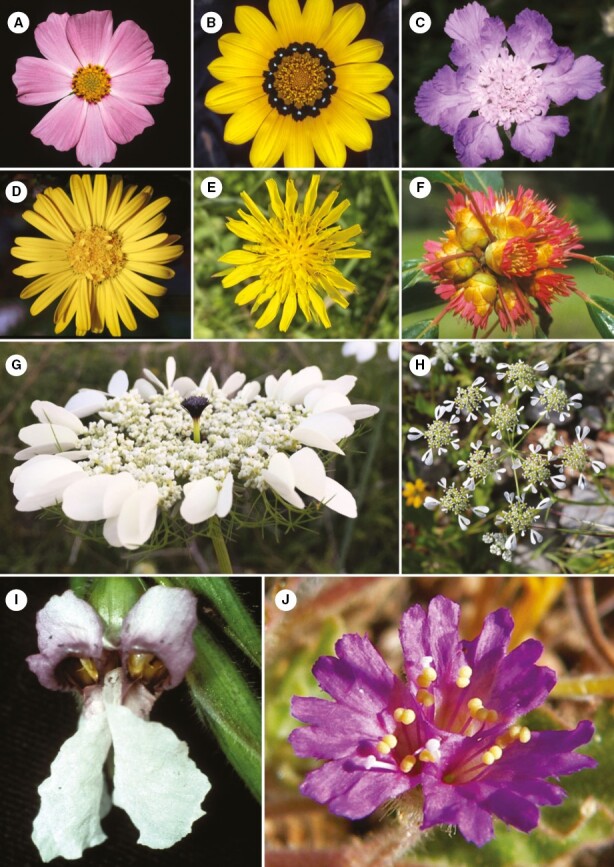
Floral pseudanthia *sensu stricto*. (A–C) Heads with actinomorphic central florets and monosymmetric ray florets: (A) *Cosmos bipinnatus* (Asteraceae); (B) *Gazania rigens* (Asteraceae); (C) *Scabiosa* sp. (Caprifoliaceae). (D) Head of headlets with monosymmetric ray flowers at the periphery of the entire blossom: *Oedera capensis* (Asteraceae). (E–F) Head with monosymmetric flowers only: (E) *Tragopogon pratensis* (Asteraceae); (F) *Rhodoleia championii* (Hamamelidaceae). (G) Umbel of umbellets with ray flowers at the margin of entire blossom: *Artedia squamata* (Apiaceae). (H) Umbel of umbellets with ray flowers at the margin of individual umbellets: *Tordylium apulum* (Apiaceae). (I) Zygomorphic blossom made of mirror-imaged pair of asymmetric flowers with showy staminodes: *Thalia geniculata* (Marantaceae). (J) Triad of monosymmetric florets forming an actinomorphic blossom: *Allionia incarnata* (Nyctaginaceae). Photos: B, E, I from Claßen-Bockhoff (2023), H, Stan Shebbs,Wikimedia Commons.

Pseudanthia with flower dimorphism are rare at the family level (Table 1) and generally restricted to only a few groups of campanulid eudicots (Asteraceae, Caprifoliaceae, Apiaceae; Fig. 3R, S, U). Interestingly, whereas some loosely aggregated inflorescences with actinomorphic flowers (Fig. 1E, F) develop advertising structures consisting of enlarged sepals, calyx or corolla lobes in peripheral position, floral pseudanthia always have monosymmetrical (zygomorphic) ray flowers with one or several petals (Fig. 6F–H) or sympetalous corolla lobes (Fig. 6A–E) as showy elements. Ray flowers usually surround a simple head (Fig. 6A–C, E, F) or umbellet (Fig. 6H), but can also develop at the margin of complex pseudanthia composed of multiple such units (Fig. 6D, G). An example of floral pseudanthia outside campanulids is the mimosoid genus *Neptunia* Lour. In some of its representatives, an inflorescence is a head (probably derived from a highly congested spike) with three types of flowers: perfect flowers occupying the distal two-thirds of the entire blossom, a narrow zone of male flowers situated just below them and a basal zone of sterile florets with showy petaloid staminodes (Tucker, 1988). Apart from *Neptunia*, *Calliandra surinamesis* Benth. has pseudanthia *sensu stricto*, whereas in other mimosoid genera with heteromorphic flowers [*Xylia* Benth., *Parkia* R.Br., *Dichrostachys* (DC.) Wight & Arn] compactness or structural subdivision is lacking.

Beyond dimorphic floral pseudanthia, there are few examples in which flower similarity is based alone on a specific arrangement of zygomorphic flowers. *Rhodoleia championi*, representing a monotypic subfamily in the Hamamelidaceae, has bell-shaped pseudanthia (Fig. 6F) 4–7 extremely zygomorphic flowers, arranged radially in a single row (Claßen-Bockhoff, 1990). The three enlarged petals of each flower are directed towards the periphery of the blossom and their stamens and styles form a homogenous centre. Pseudanthia of *Heteranthera gardneri* (Hook.f.) M.Pell. (Pontederiaceae) and *Thalia geniculata* L. (Marantaceae; Fig. 6I) are formed by two mirror image flowers (Rutishauser, 1983; Claßen-Bockhoff, 1991*b*). They are monosymmetrical in the former and asymmetrical in the latter resulting in an actinomorphic disc blossom and zygomorphic lip blossom, respectively. Additionally, actinomorphic pseudanthia comprising three zygomorphic flowers (Fig. 6J) can be found in *Allionia* L. (Nyctaginaceae).

### Underlying organization

Pseudanthia cannot be unequivocally linked to a particular type of reproductive shoot architecture (Table 1). Proceeding from the view that all pseudanthia are modified inflorescences, Claßen-Bockhoff (1990) formally classified different types of pseudanthial architectures with respect to the absence or presence of a terminal flower, the branching pattern of the inflorescence, and the presence or absence of extrafloral bracts or ray flowers. Based on her analysis, pseudanthia are common in head-shaped blossoms which lack (Fig. 5U) or have common receptacles (as in Asteraceae; Fig. 6A, B, D, E). More rarely, spikes (Fig. 5A), a cluster of spadices (Fig. 5B) or spikelets (Fig. 5C), cymes (Fig. 5D), thyrses (and thyrse-like floral units, see Fig. 5H–J), or cyathia (Fig. 5W, X) are found. In some families, pseudanthia are formed by pattern repetition on a more complex organizational level. Examples occur in Asteraceae (Figs 5M, 6E), Apiaceae (Figs 5V and 6F) and Bruniaceae (*Brunia fragarioides* Willd., Claßen-Bockhoff, 2000), but also in the monocot lineage Eriocaulaceae (Stützel & Trovó 2013).

**Fig. 7.**
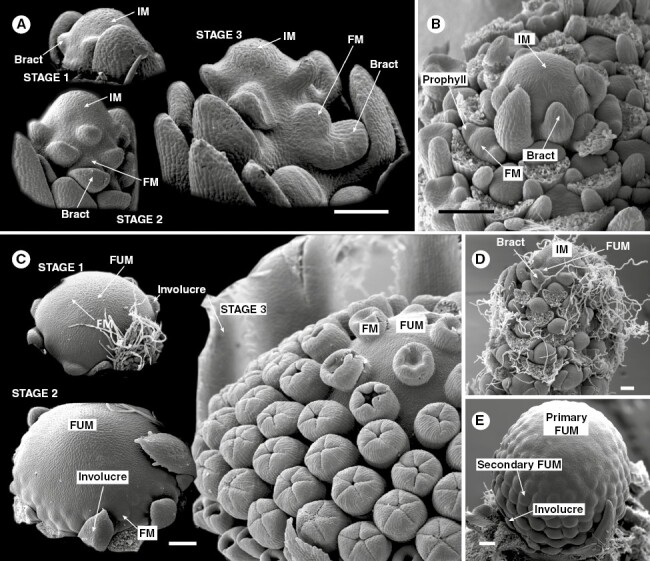
Inflorescences and floral units. (A, B) Inflorescence meristems with flowers: (A) *Veronica albicans* (Plantaginaceae): three developmental stages of the raceme meristem showing the acropetal segregation of bract-subtended flower meristems; (B) *Actinodium cunninghamii* (Myrtaceae, see Fig. 5S for mature blossom): head meristem with floral meristems. (C) Floral unit meristem with flowers: *Calendula officinalis* (Asteraceae): three developmental stages of the capitulum meristem with flower meristems (stages 1 and 2) and young bract-less florets (stage 3). (D) Inflorescence meristem with floral units: *Ligularia stenocephala* (Maxim.) Matsum. & Koidz. (Asteraceae): bract-subtended capitulum meristems (FUM) in a raceme (IM) occupying flower position. (E) Floral unit meristem with floral units: *Echinops bannaticus* Rochel ex. Schrad.: headlet meristems fractionating from a head meristem. Abbreviations: FUM – floral unit meristem, IM – inflorescence meristem, FM – floral meristem. Photo: B, from Claßen-Bockhoff *et al.* (2013).

In recent years, ontogenetic studies have revealed that numerous pseudanthia do not develop from inflorescence meristems. They, instead, fulfil the definition of floral units *sensu*Claßen-Bockhoff & Bull-Hereñu (2013) originating from expanding, determinate meristems (floral unit meristems, FUMs) with flower-like qualities (Fig. 7). As of now, floral units have been thoroughly investigated in Asteraceae (Harris, 1995; Zhang & Elomaa, 2021), Apiaceae (Baczyński *et al.*, 2022a), Nyssaceae (Claßen-Bockhoff and Arndt, 2018; Gong *et al.*, 2018) and Euphorbiaceae (Prenner and Rudall, 2007; Prenner *et al*., 2011; Claßen-Bockhoff and Frankenhäuser, 2020), but they can be also identified in other lineages with pseudanthial representatives, including Saururaceae (Tucker, 1981, 1985; Fig. 5A), Araceae (Barabé and Lacroix 2008*a*, *b*), Eriocaulaceae (Stützel & Trovó, 2013), Hamamelidaceae (Claßen-Bockhoff and Bull-Hereñu, 2013), Rutaceae (Claßen-Bockhoff *et al.*, 1991), Moraceae (Leite *et al.*, 2021), Cornaceae (Liu *et al.*, 2013; Fig. 5H), Dioscoreaceae (Caddick *et al.*, 2000) and Caprifoliaceae–Dipsacoideae (Naghiloo and Claßen-Bockhoff, 2017; Fig. 6D).

An interesting (still unpublished) pattern of pseudanthium formation has been found in the South African genera *Staavia* Dahl (Bruniaceae) and *Euchaetis* Bartl. & H.L.Wendl. (Rutaceae). *Staavia dodii* Bolus (Fig. 5T) and *Euchaetis longibracteata* (Fig. 5U) share head-like blossoms of similar shape, size and symmetry. In both species, the terminal flower is the first to bloom, followed by bilateral flowers arising basipetally (for *Staavia* see Claßen-Bockhoff, 2000). In contrast to the basipetal flowering sequence, such a completely basipetal initiation of flowers is rare among pseudanthia.

The examples of *Actinodium cunninghamii* (Myrtaceae, inflorescence with modified short shoots; Fig. 5S), *Myriocephalus helichrysoides* A. Gray (Asteraceae, secondary floral unit with showy bracts; Fig. 5M) and *Staavia dodii* (Bruniaceae, basipetally initiated cluster of flowers with showy bracts; Fig. 5T) clearly demonstrate that similar flower-like patters evolved repeatedly based on different meristem conditions. Thus, structural diversity is much higher than the figural similarity may suggest.

## ECOLOGICAL PATTERNS IN PSEUDANTHIA

Pseudanthia can be described by a character syndrome with density, smallness and division of labour being the most important traits. These characters evolve independently from each other as documented by the multiple examples of modified inflorescences (Fig. 1). Taking the Asteracean capitulum as the prototype of a pseudanthium, it is clear that the combination of all three characters results in unambiguous flower-like similarity. If the ray flowers or showy bracts dominate the blossom, large (*Congea* Roxb, Fig. 5D; *Bougainvillea* Comm. ex Juss., Fig. 5E, Nyctaginaceae) or loosely arranged florets (*Xanthosia*, Fig. 5V) do not hinder its overall flower-like appearance. However, the gradual transition between modified inflorescences and pseudanthia makes the grouping subjective. This is the reason why we focus on clear examples here and do not insist on a sharp definition.

### Degree of flower similarity

Leppik (1969) reconstructed the evolution of angiosperm flower forms in different lineages and found actinomorphic, cup-shaped flowers with unstable numerical patterns to be ancestral, followed by those with a fixed number of organs, stereomorphic and finally monosymmetrical flowers. According to him, this evolutionary trajectory, coupled with increased synorganization and specialization, created the present diversity of angiosperm flowers. Interestingly, within core eudicots, the formation of asteracean heads somehow ‘reversed’ the trend of floral specialization. While individual florets of composites are highly derived, the entire blossom recapitulates conditions of ancestral flowers in being actinomorphic and generalistic in terms of pollination. Indeed, floral miniaturization and aggregation may present a chance for despecialization, creating new adaptive options (Claßen-Bockhoff, 1992) including shifts from anemophily to generalized zoophily as described for some Cyperaceae (Villa-Machío *et al.*, 2020; da Costa *et al.*, 2021).

Leppik (1969) proceeded from the view that pollinator-mediated selection is crucial for pseudanthia formation. Once adapted to a certain flower form, pollinators would eagerly visit similar-shaped blossoms, irrespective of them being a single flower or flower aggregate. The most obvious example of this morphological convergence is the functional division of pseudanthia into a central part that performs reproductive function and a peripheral, sterile part that serves pollinator attraction. The importance of visual cues from showy bracts/ray flowers has been attested to in numerous studies, including both observations (Andersson, 1996; Armbruster *et al.*, 2005; Song *et al.*, 2012; Pérez‐Barrales *et al.*, 2013) and manipulation experiments (Celedón-Neghme *et al.*, 2007; Song *et al.*, 2012) and at least in some species their effect seems dependent on the overall size of the floral display and abundance of pollinators (Andersson 1999, 2008).

Numerous pseudanthia seem well suited for generalist pollinators. They are frequently whitish/yellowish, actinomorphic, flat or cup-shaped, and similar in size to medium to large flowers (2–4 cm). However, the form of the pseudanthium and its flowers may diverge considerably; that is, generalistic-looking heads may include long-tubed flowers adapted to butterflies or long-tongues flies. *Bougainvillea* species (Fig. 5E), with a funnel-formed pseudanthium and narrow-tubed sphingophilic flowers, demonstrate that adaptation to specialized pollinators is due to flower rather than blossom form.

Some pseudanthia underwent a profound morphological and ecological specialization linked to a high degree of synorganization, creating unique forms such as bat-pollinated bell-blossoms (Amorim *et al.*, 2021) or deceptive traps known from Araceae (Gottsberger and Amaral, 1984; Bernhardt, 2000). The adaptation to certain groups of pollinators has ultimately led to several independent shifts from actinomorphy to monosymmetry in pseudanthia – a common and well-recognized evolutionary transition found in single flowers (Reyes *et al.*, 2016). The zygomorphic blossoms of *Dalechampia* Plum. ex L. (Euphorbiaceae) present resin as reward for euglossine bees (Armbruster and Webster, 1979) and some of them [i.e. *D. spathulata* (Scheidw.) Baill., Fig. 5J] develop perfume blossoms with a morphologically derived oil-secreting centre (Froebe *et al.*, 1983). The floral pseudanthium of *Thalia geniculata* (Fig. 6I) forms a zygomorphic blossom adapted to pollination by *Xylocopa* (Latreille, 1802) bees (Claßen-Bockhoff, 1991*b*), while some hummingbird-pollinated *Euphorbia* species (e.g. *E. tithymaloides* L., *E. macrocarpa* Boiss. & Buhse.; Fig. 5X) develop tube-like blossoms with separate nectar chambers using involucral excrescences (Michaelis, 1924; Dressler, 1957).

A relatively large number of pseudanthia are bird-pollinated (Porsch, 1923), showing traits such as reddish or bright red coloration, production of a large amount of low-concentration nectar and stability necessary for their passerine and honeyeater pollinators (Keighery, 1975; Gu *et al.*, 2010). Based on blossom construction, two different forms of ornitophilous pseudanthia can be distinguished. Bell-shaped blossoms tend to have a homogeneous centre which accumulates nectar from all flowers, creating a huge supply for visiting birds. Claßen-Bockhoff (1992) postulated that the evolution of such architecture in *Darwinia* (Myrtaceae) Raf. species and *Pimelea physodes* Hook. (Thymeleaeceae, Fig. 5P, Q; Keighery, 1975) might have been a side effect of bract enlargement necessary for bud protection in mountainous habitats. Heads, in contrast, may provide pollinators with a landing place on top of the flowers. In *Etlingera elatior* (Fig. 5K), the inflorescence axis elongates with the acropetal flowering sequence, maintaining a constant distance between the sitting bird and nectar reward (Classen, 1987). Additionally, each open flower presents a strong visual cue in the form of a contrasting yellow margin which resembles the open bill of a young bird.

Many pseudanthia are bicoloured as the showy bracts differ in colour from the centre (e.g. Fig. 5J, L, M). In some Apiaceae, white umbels present a black centre (Fig. 6G) which has been postulated to create a ‘fly catcher’ effect (Eisikowitch, 1980) or other form of insect mimicry (Goulson *et al.*, 2009). Unlike flowers, nectar guides (in the human visual spectrum) are largely lacking in pseudanthia. Exceptions are found in some heteromorphic heads of Asteraceae with ring-like patterns overarching all ray flowers, or a colour contrast between disc and ray flowers. These visual cues probably play a role similar to the UV-patterns found in some species (Burr *et al.*, 1995). An exciting example of flower-like specialization in pseudanthia evolved in *Gorteria diffusa* Thunb. (Asteraceae). Its ray flowers produce a distinct bullseye pattern (Ellis *et al.*, 2014) in the form of dark spots which act as a sexual deception for its main pollinator *Megapalpus capensis* (Wiedeman, 1828). Thomas *et al.* (2009) also found that those apparently randomized spots reflected the divergence angle during flower meristem initiation.

When compared against the background of ‘true flowers’, pseudanthia constitute a remarkable example of convergent evolution in many angiosperm lineages. Their repeated acquisition in the highly derived Asteridae alone (when analysed on the level of species/genera) can be seen as support for Leppik’s view of *pseudanthic recapitulation.* However, note that the term *recapitulation* is slightly misleading as it suggests that pseudanthia arose repeatedly only by convergence and excludes the view that developmental constraints such as meristem changes may have paved the way towards pseudanthia formation as a predisposition.

### Selective advantages of small-flowered aggregates

Good (1956) held the view that pseudanthia formation was the result of an *aggregation tendency* closely linked to flower miniaturization and ultimately ending in the emergence of flower-like units. However, he only briefly discussed the selective advantages and developmental conditions triggering aggregation in angiosperms.

Densely packed aggregates of small flowers are a common phenomenon across angiosperms. Both features affect the pollination and breeding systems of the plant. Species with flat blossoms, including simple flowers, tend to be ecological generalists (i.e. many Apiaceae or Asteraceae) providing food or other rewards to diverse flower visitors. They rarely suffer from pollinator limitation, allowing them to colonize new habitats. In other plant lineages, floral aggregation probably arose with a switch to wind (Linder and Rudall, 2005) or rodent pollination (Rourke, 1998; Johnson *et al.*, 2001; Kleizen *et al.*, 2008).

Small flowers are cheap in investment and usually have a small number of ovules. Being aggregated, they can compensate for a short flowering time by the large number of flowers. If the aggregate is surrounded by ray flowers or showy bracts, ephemeral flowers benefit from the enduring attraction of these structures. The prolonged presentation of many few-ovuled flowers is beneficial for pollination as it may lead to higher rates of outcrossing compared to single flowers with many ovules (Burtt, 1961). Indeed, according to our investigation, the majority of pseudanthial plants with highly aggregated blossoms (especially among eudicots) produce flowers with a single or relatively sparse (<10) number of ovules (Table 1).

Aggregation is entwined with evolution of diverse reproductive systems found in flowering plants. Pollinator movements on condensed, flat-surfaced inflorescences are less consistent than on loosely aggregated and three-dimensional ones, which increases pollen discounting (loss of pollen available for outcrossing) and the chances of geitonogamy (Hardegger and Sturm, 1998; Jordan, 2000; Ishii *et al.*, 2008). When miniaturization makes it hard to avoid self-pollination by physically separating male and female function within florets, a plant is forced to regulate it on the level of an entire inflorescence through either temporal (protandry, protogyny) or spatial (andromonoecy, androdioecy, gynomonoecy, gynodioecy) flower-phase segregation (Plitmann, 1995).

The expression of breeding systems is precisely controlled via resource partitioning (Charlesworth and Charlesworth, 1978; Saur Jacobs and Wade, 2003; Goldberg *et al.*, 2017) that can additionally fuel the process of pseudanthic recapitulation. In andromonoecious Apiaceae, the number of staminate flowers generally declines with increasing order (and decreasing size) of the produced umbels which implies that in these plants, the reproductive outcome is predominantly controlled by a variable investment in male function (Spalik, 1991; Schlessman and Graceffa, 2002; Schlessman *et al.*, 2004; Reuther and Claßen-Bockhoff, 2010). Due to the centripetal developmental sequence, resources are first allocated to peripheral floral meristems that develop into hermaphrodite flowers. Such increased nourishment of marginal florets can potentially promote their evolution into ray flowers. Bawa and Beach (1981) hypothesized that the prevalence of gynomonoecy in Asteraceae can result from evolution of showy rays, as resources that could be otherwise allocated to stamens in marginal florets are spent on enlargement of petals. Although tentative, this explanation is not easily applicable to all composites, as both homogamous and heterogamous heads consisting entirely of monoclinous flowers occur in this family (Kilian and Gemeinholzer, 2009; Torices and Anderberg, 2009).

The distribution of the reproductive organs in individual flowers is remarkably constant. Carpels are almost inevitably surrounded by stamens. While numerous pseudanthia recapitulate this pattern, the arrangement of staminate and carpellate flowers in blossoms is not as highly constrained. Consequently, pseudanthia combine the selective advantages of flower-like patterns for pollinator attraction with developmental plasticity of inflorescences for shaping breeding systems. For example, in Euphorbiaceae, spurges mimic the organization of a typical flower (cyathia composed of carpellate flower surrounded by staminate ones), whereas in *Dalechampia* staminate flowers are those that occupy the central position within the blossom (Prenner and Rudall, 2007; Gagliardi *et al.*, 2018). Pseudanthia of Eriocaulaceae are frequently arranged so that the few staminate flowers develop in the median part of the head (Stützel, 1998). In many Asteracean heads, the outermost flowers are carpellate. They open first, increasing the chance of outcrossing and making capitula an equivalent of protogynous flowers (despite individual disc florets being strongly protandrous). Even blossoms of *Lacandonia schismatica* and *Trithuria*, usually considered to be the only examples of inside-out flowers, have been interpreted as pseudanthia by some authors (Rudall, 2003; Rudall *et al.*, 2016).

Apart from pollination biology, blossoms may aggregate in response to abiotic conditions. For example, secondary heads in Asteraceae–Nassuavinae could have evolved in response to colonization of arid habitats, as enlarged flower-subtending receptacles may function as an additional water storage organ (Katinas *et al.*, 2008). Moreover, inflorescence condensation creates an opportunity to transfer protective function from sepals of individual flowers to bracts that can later evolve into advertising structures. The aggregation of (partial) inflorescences in some Bromeliaceae allows for flower enclosure by enlarged bracts. These serve as canisters for rainwater, which is necessary for heat transfer and proper floral development (Nogueira *et al.*, 2019, 2021), but also create showy display for pollinators. Similar elongated bracts evolved in some Himalayan genera of Asteraceae, such as *Cremanthodium* Benth. (Chen *et al.*, 2013) and *Saussurea* DC. (Tsukaya *et al.*, 2002; Yang and Sun, 2009), to preserve heat and protect pollen from UV radiation. The dual protective and attractive function of enlarged bracts was also confirmed for *Davidia involucrata* L. (Sun *et al.*, 2008) and *Rheum nobile* (Song *et al.* 2012).

## EVOLUTIONARY PATHWAYS: AGGREGATION VS. CHANGE IN MERISTEM QUALITIES

Recently, it has become clear that a purely typological classification of reproductive shoots hinders our understanding of their diversity and evolution. Although detailed molecular studies of selected plants (Castel *et al.*, 2010; Bartlett and Thompson, 2014; Périlleux *et al.*, 2014) were helpful in recognizing the basic principles of branching and meristem determinacy, an incorrect application of confounded morphological terminology and a model-oriented approach makes it hard to properly interpret structures that deviate from these patterns. Therefore, dense flower clusters are usually interpreted as condensed inflorescences. However, they can also result without aggregation from profoundly altered meristems, for example when the reproductive meristem merges into a floral unit meristem with flower-like qualities. The finding that pseudanthia can originate from different types of reproductive meristems (Fig. 7) results in two hypothetical explanations for their evolution, the aggregation and meristem change theories.

### Aggregation theory

**Fig. 8.**
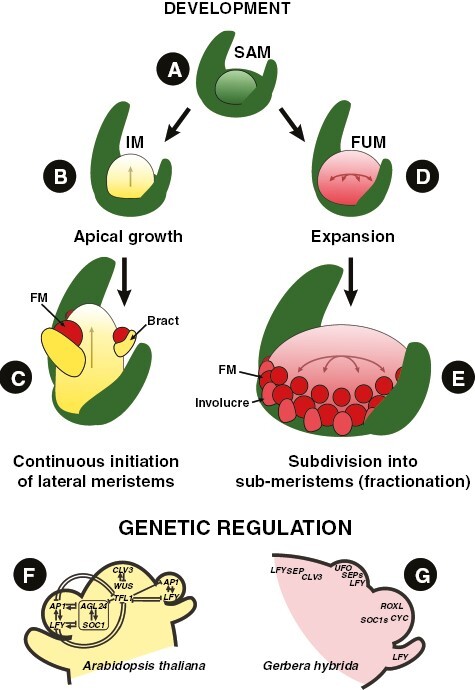
Development of IMs and FUMs. (A) Indeterminate SAM. (B) SAM transforms into an IM showing continuous (but limited) apical growth. (C) IM sequentially initiates (segregates) lateral meristems. (D) SAM transforms into a determinate FUM that grows with expansion. € FUM rapidly subdivides (fractionates) into new sub-meristems. (F) Regulation of raceme development in *Arabidopsis thaliana* (based on Davila-Velderrain *et al.*, 2016; C. Liu *et al.*, 2009; Ratcliffe *et al.*, 1999; Valentim *et al.*, 2015). The upregulation of *TFL1* and maintenance of the regulatory loop between *WUS* and *CLV3* allows for continuous growth of the inflorescence apex through stem-cell activity. (G) Regulation of head development in *Gerbera hybrida* (based on Zhao *et al.*, 2016; Zhang and Elomaa, 2021; T Zhang *et al.*, 2021). Determinate FUMs show an expanded domain of *CLV3* expression, as well as uniform expression of *SEP* and *LFY* orthologues in the mantle zone at the incipient developmental stages. Abbreviations: FUM – floral unit meristem, IM – inflorescence meristem, FM – floral meristem.

Inflorescence meristems (Figs 7A, B and 8B, C, F) are characterized by acropetal segregation of inflorescence elements. Internodes are elongated late in development and shape the architecture of the inflorescence. The first theory for formation a pseudanthium from an inflorescence meristem is thus internode inhibition, called aggregation. Maresquelle (1970) and Sell (1976) illustrated series of increasing aggregation in selected angiosperm lineages. Pozner *et al.* (2012), likewise, interpreted the capitulum of the Asteraceae as an aggregated inflorescence having originated from an ancestral thyrse. However, as recently proven, the capitulum does not develop from an inflorescence but from a floral unit meristem (Figs 7C, E and 8D, E, G; Zhang and Elomaa 2021).

According to the transient model (Prusinkiewicz *et al.*, 2007), inflorescence development is controlled by the level of a *vegetativeness* (*veg*) factor which declines as indeterminate shoot apical meristems (SAMs) segregate lateral meristems (branches). When the level of *veg* becomes sufficiently low, lateral meristems transform into determinate floral meristems. This simple model can be superimposed onto the flowering-time gene regulatory network (FTGRN, see Davila-Velderrain *et al.*, 2016; C. Liu *et al.*, 2009; Ratcliffe *et al.*, 1999; Valentim *et al.*, 2015). In *Arabidopsis thaliana* (Fig. 8F), a high level of *veg* corresponds to the expression of the shoot identity gene *TERMINAL FLOWER 1* (*TFL1*), a low level to the expression of the flower identity gene *LEAFY* (*LFY*), and the transient stage (branching) to the expression of conserved flowering-time regulators such as *SUPRESSOR FOR OVEREXPRESSION OF CONSTANS* (*SOC1*), *SHORT VEGETATIVE PHASE* (*SVP*), *AGAMOUS-LIKE 24* (*AGL24*) and *XANTAAL2* (*XAL2*). Although the rapid decline of *veg* (and alterations to FTGRN in general) can explain the origin of some highly aggregated inflorescences (Azpeitia *et al.*, 2021), the transient model cannot be easily applied to floral units due to different meristem conditions.

### Meristem change theory

FUMs (Figs 7C, E and 8D, E, G) are characterized by determinacy, understood as the lack of stem-cell activity (not to be confused with ‘openness’ or the lack of terminal flowers, which may or may not develop into floral units depending on available space; see Bull-Hereňu & Claßen-Bockhoff, 2010). Consequently, flower primordia are not continuously segregated from the meristem apex, but instead arise by the process of fractionation that subdivides the available meristem until the entire surface of the FUM is used (Claßen-Bockhoff & Bull-Hereñu, 2013; Claßen-Bockhoff, 2016). Apart from the difference in size, FUMs share most qualities with flower meristems (FMs), which might explain their numerous flower-like genetic and developmental patterns (Broholm *et al.*, 2008; Carlson *et al.*, 2011; Zhao *et al.*, 2016; Baczyński *et al.*, 2022*a*).

Our knowledge about the genetic patterning of FUMs comes predominantly from studies conducted on their most notable example: flower-heads of Asteraceae (Fig. 8G; Elomaa *et al*., 1993). The greatest difference between typical Astearcean FMs and FUMs is their initial bulk. Flower-head meristems can reach a few millimetres in diameter (as in sunflower), while FMs rarely exceed a tenth of this size. Additionally, both meristems are patterned by the same process of subapical expansion and fractionation controlled by gradients of auxins established by transmembrane localized exporters belonging to the PIN-FORMED (PIN) protein family (Zhou and Luo, 2018). Disruption of these natural auxin flows can lead to severe changes in the morphology of both flowers (Cheng and Zhao, 2007) and floral units. Exogenous application of IAA (indole-3-acetic acid) onto developing flower-heads of *Matricaria inodora* L. (Zoulias *et al.*, 2019) leads to bracts or ray flowers developing in the centre of the unit. A similar morphogenetic alteration can be achieved by mechanical force, as proven by historical experiments on sunflower meristems (Palmer and Marc, 1982; Hernandez and Palmer, 1988). Recent analyses conducted on *Gerbera* L. indicate that wounding can disrupt the expression of its *CLV3* orthologue (*GhCLV3*), causing auxin-mediated re-patterning of the capitulum. These results constitute a substantial background for future studies, which will probably aim to disentangle the relationships between PIN-mediated transport, local biosynthesis of auxins and development of FUMs.

Although capitula lack a terminal flower, the uniform expression of the LFY orthologue (*GhLFY*) in the early flower-head meristem of *Gerbera* defines it as a determinate (lacking stem cell activity) structure, similar to an FM. The suppression of *GhLFY* results in an altered FUM, the centre of which is never fully consumed by flowers or, in the case of strong transgenic lines, a complete loss of flower identity and their substitution with bract-like organs (Zhao *et al.*, 2016). Interestingly, MADS-box *SEPALLATA*-like genes (*SEP*), which similarly to *LFY* account for identity of FMs in *Arabidopsis* (Pelaz *et al.* 2000), underwent duplication and neofunctionalization to control the determinacy of both flowers and entire head of Asteraceae (Zhang *et al.*, 2017). *Gebera*’s orthologue of *UNUSUAL FLORAL ORGANS* (*UFO*), *GhUFO*, is yet another gene involved in patterning of the capitulum, but its function is conserved and related to FM identity. Silencing of *GhUFO* converts the asteracean floral unit into a structure reminiscent of a single flower with multiple organs initiated in a whorl-like pattern (Zhao *et al.*, 2016). These results indicate that while *LFY* and *SEP* are implicated in the early patterning of FUM, their co-expression with *UFO* is required to specify future florets. When expression of *UFO* is lacking, flower-heads assume the developmental trajectory of a single flower, what can be seen as support for a peramorphic origin of FUMs (Claßen-Bockhoff & Frankenhäuser, 2020; Baczyński *et al.*, 2022).

The association between FUs and pseudanthia is very clear. However, without further research it is impossible to confirm if acquisition of the former constitutes an important prerequisite for evolution of the latter. Various forms of showy bracts are common in flowering plants, including not only highly condensed (Table 1) but also relatively loosely aggregated inflorescences in which individual flowers constitute a basic pollination unit (Fig. 1). Such petaloid phyllomes are more likely to form in aggregated blossoms, but are not necessarily linked to floral units. On the other hand, flower dimorphism including ray flower formation is generally restricted to campanulids and evolved independently numerous times in three of their major clades – Asteraceae (C Zhang *et al.*, 2021), Apiaceae (Baczyński *et al.*, 2022), Caprifoliaceae–Dipsacoideae (Carlson *et al.*, 2011; Panero *et al.*, 2014). In each of these groups, floral units pre-date ray flowers, suggesting that unique qualities of floral unit meristems may drive the evolution of enlarged marginal florets.

## GENETIC PATTERNING OF RAY FLOWERS AND SHOWY BRACTS

Pseudanthia arose independently in dozens of angiosperm lineages, but the genetic mechanisms underlying the evolution of ray flowers and showy bracts may be remarkably homogeneous. The patterning of ray flowers has been thoroughly studied in Asteraceae (Fig. 8G). Their identity is established primarily by TCP genes: a family of plant-specific transcription factors, which play a crucial role in environment-mediated growth responses (Danisman, 2016). The sequence of the TCP domain encodes a DNA-binding bHLH motif, which was initially identified in maize (*TEOSINTE BRANCHED1*, *TB1*), snapdragon (*CYCLOIDEA*, *CYC*) and rice (*PROLIFERATING CELL FACTOR1* and *PROLIFERATING CELL FACTOR*2, *PCF1* and *PCF2*) (Cubas *et al.*, 1999). Eudicot TCP genes are subdivided in two classes. The TCP I class encompasses orthologues of *PCF* genes, whereas the TCP II class includes orthologues of *TB1* and *CYC* genes (Martín-Trillo and Cubas, 2010). The canonical function of *CYC* is related to establishment of monosymmetry in individual flowers (Luo *et al.*, 1996); however, in Asteraceae, *CYC-*like genes underwent several duplications, creating multiple paralogues that subsequently neo-functionalized creating differential expression patterns in central and peripheral florets. Overexpression of the *GhCYC2* gene in *Gerbera* (the transcript of which is normally found in the ligule of ray flowers) transforms central radially symmetrical disc flowers into bilaterally symmetrical units similar to those found on the periphery of the capitulum (Broholm *et al.*, 2008; Tähtiharju *et al.*, 2012; Juntheikki-Palovaara *et al.*, 2014). Gene expression patterns recovered in *Senecio vulgaris* L. indicate that its three CYC2 clade genes regulate ray flower development in a similar manner, promoting growth of the ventral ligule (Garcês *et al.*, 2016). Interestingly, CYC2 genes had independently expanded in Apiaceae (Baczyński *et al.*, 2022) and Caprifoliaceae–Dipsacoideae (Carlson *et al.*, 2011; Berger *et al.*, 2016) and their paralogues also show differential expression in central and ray florets.

The development of showy bracts has been linked to heterotopic expression of MADS-box genes, including orthologues of class B genes *APETALA3* (*AP3*) and *PISTILLATA* (*PI*) from *A. thaliana*, which canonically take part in the establishment of identity of petals and stamens (Theißen *et al.*, 2000; Becker and Theißen, 2003). In pseudanthial dogwoods (*Cornus* L., Fig 5H), petaloid bracts evolved independently in the so-called DW (dwarf) and BB (big-bracted) groups with important differences visible not only in the bract position but also in the expression of B-class orthologues during their morphogenesis (Zhang *et al.*, 2008; Feng *et al.*, 2012). The handkerchief-like semaphylls of *Davidia involucrata* are also patterned with the aid of B-class and C-class MADS-box genes and might have evolved to compensate for apetaly in Nyssaceae (Manchester *et al.*, 1999; Vekemans *et al.*, 2012). Outside Cornales, the MADS-box-related organ petaloidy has been inferred for nectariferous bracts of *Marcgravia* L. (Marcgraviaceae; Geuten *et al.* 2006) that show heterotopic expression of E-class *SEPALLATA*-like genes during development (Pelaz *et al.*, 2000).

## CONCLUSIONS AND FUTURE PROSPECTS

This review summarizes the historical and current concepts on the structure, form and function of pseudanthia and provides their clear operative definition, by narrowing the term to structurally subdivided flower-like blossoms (or pseudanthia *sensu stricto*). Its overarching goal, however, is to give an overview of the phylogenetic and phenotypic diversity of pseudanthia and evoke a broader interest in these fascinating structures, as until now they have rarely been recognized as a distinct evolutionary phenomenon. This matches the prevalent ‘floricentrism’ (Harder *et al.*, 2004) and general disinterest in inflorescences. What adds insult to injury is that some of the largest pseudanthial families (i.e. Asteraceae or Apiaceae) are renowned for their highly generalized, ‘boring’ floral morphology and promiscuous pollinator interactions which are notoriously difficult to study due to seasonal and geographical variability (Dellinger, 2020). However, considering that pseudanthia recapitulate some well-established morphological patterns recognized in single flowers, they may provide unique insights into the evolution of not only generalized but also specialized pollination syndromes. Manipulative experiments, i.e. addition/removal of showy bracts or alteration of merism/symmetry, can potentially provide links between the resource partitioning, architecture and pollination biology of pseudanthia. In addition to ecology, pseudanthia constitute a fascinating subject for macroevolutionary studies, as factors contributing to their evolvability are virtually unknown. Moreover, the fact that pseudanthia and other forms of modified inflorescences seem associated with spectacular instances of adaptive radiation (i.e. Asteraceae, *Euphorbia*) indicates that aggregated, flower-like blossoms, at least in some instances, can be considered as potential key evolutionary innovations.

Reproductive shoots are usually regarded only as mature structures (and the source of auxillary traits in taxonomic studies) but to fully understand the evolution of pseudanthia, we need to focus on their basic patterning mechanisms. The development of inflorescences has as yet been sufficiently described only in selected model species, such as *Arabidopsis* or *Petunia*. With formal recognition of floral units (Claßen-Bockhoff and Bull-Hereñu, 2013) and other potentially new types of lineage-specific reproductive modules (Zhong and Kong, 2022), it has become clear that additional morphological and ontogenetic data from distantly related angiosperm groups are crucial for our understanding of this diversity. Campanulid eudicots constitute a good candidate system for evo-devo studies on pseudanthia, as several lineages in this speciose clade independently acquired FUMs, as well as pseudanthia with showy ray flowers or extrafloral elements. Moreover, transformation protocols (Elomaa *et al.*, 1993; Knittel *et al.*, 1994; Hardegger and Sturm, 1998; Baranski, 2008; Klimek-Chodacka *et al.*, 2018; Yildirim *et al.*, 2022) and genomic resources (Iorizzo *et al.*, 2016; Badouin *et al.*, 2017) that could facilitate gene discovery and functional studies, are already available for selected taxa.
